# Anatomical Study of the Ultrasound-guided Percutaneous Tenotomy of the Iliopsoas Muscle Tendon in Cadavers: A Feasible Technique?

**DOI:** 10.1055/s-0044-1790215

**Published:** 2025-04-11

**Authors:** Caio Ikuhara Gonçalves, Nayra Deise dos Anjos Rabelo, Walter Ricioli Junior, Marco Rudelli, Giancarlo Cavalli Polesello

**Affiliations:** 1 Grupo de Quadril, Departamento de Ortopedia e Traumatologia, Faculdade de Ciências Médicas da Santa Casa de São PauloSão Paulo, SP, Brasil

**Keywords:** cadaver, hip, hip joint, tenotomy, ultrasonography

## Abstract

**Objective** To evaluate the efficacy and safety of percutaneous tenotomy of the iliopsoas muscle tendon guided by ultrasound (US) in cadavers.

**Methods** We conducted an anatomical and descriptive study of the US-guided percutaneous tenotomy technique for the iliopsoas muscle tendon to review our experience performing it and its reproducibility in the clinical practice.

**Results** Of the 20 tenotomies, 17 were total, at the level of the upper edge of the acetabulum, while 3 were partial. One procedure resulted in a partial injury to the femoral nerve. We measured the distance between the place of blade introduction and the femoral nerve, a noble structure potentially at a higher risk during the procedure; the mean distance was of 8.4 mm.

**Conclusion** Iliopsoas tendon release procedures guided by US in a cadaveric model are feasible and consistently result in the total release of the tendon, except in cases of obesity, with minimal repercussions on adjacent structures, and their completion requires approximately 4 minutes.

## Introduction

Total hip arthroplasty (THA) is a widely used intervention to restore function and alleviate pain in patients with several hip joint conditions. However, even with THA success, some patients remain with persistent postoperative pain, often related to friction of the iliopsoas muscle tendon against the anterior edge of the acetabular component. 1 2 This friction, known as psoas impingement, results in inflammation, discomfort, and significant functional limitation for the patient, including going up or down stairs, getting in and out of cars, or in situations requiring hip extension. 3 An alternative before reviewing the acetabular component is an arthroscopy with an intra-articular approach and capsule opening to access the iliopsoas muscle tendon and perform a tenotomy.

The percutaneous technique may present additional advantages, such as shorter procedure time and a single incision compared with the techniques currently described, which range from arthroscopic approaches to open surgeries. 4 5

We devised an even less invasive technical variant, locating the iliopsoas muscle tendon by ultrasound (US) imaging at the impingement point and performing guided tenotomy as previously described by Sampson et al. 6 This technique is an alternative to arthroscopic surgery, which is minimally invasive but may pose more risks, including technique-related complications or irrigation fluid leakage into the iliopsoas muscle sheath. 5

We analyzed the approach, potential risk for noble structures, and the effectiveness of tenotomy in cadavers. The investigations included a meticulous evaluation of the interventional technique with a special focus on minimizing damage to surrounding noble structures.

The present study aimed to assess the efficacy and safety of percutaneous US-guided tenotomy of the iliopsoas muscle tendon in cadavers.

## Materials and Methods

This anatomical and descriptive study was developed at the Orthopedics and Traumatology Department of Faculdade de Ciências Médicas Santa Casa de Misericórdia de São Paulo and performed at the São Paulo Capital Death Verification Service (Serviço de Verificação de Óbitos da Capital, SVOC, in Portuguese) of Universidade de São Paulo (USP), with duly registration of the study team under letter number 18/2024 per its regulations and approval from the institutional Ethics Committee (CAAE number 76524523.4.0000.5479). Those responsible signed a free and informed consent form.

The study included a sample of 10 cadavers, with 20 hips not embalmed with formaldehyde.

### Tenotomy Technique

**Fig. 1:**
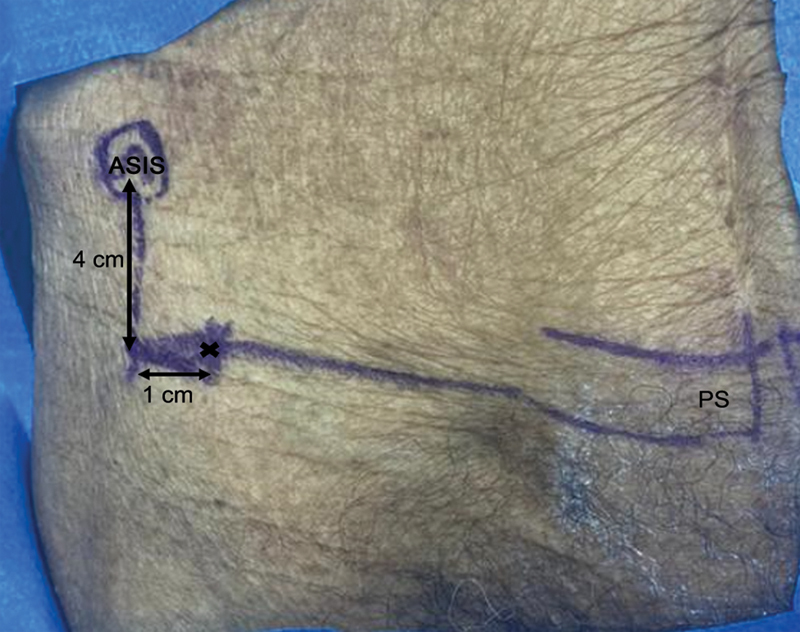
Location of the incision location, 4 cm distal and 1 cm medial from the anterosuperior iliac spine (ASIS). **Abbreviation:** PS, pubic symphysis.

**Fig. 2:**
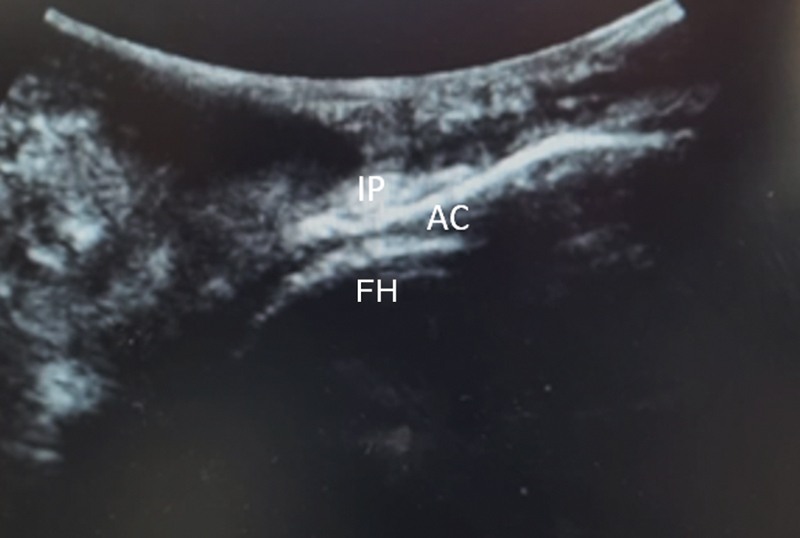
Ultrasound view of the “stacking sign” with the transducer transverse to the frontal plane showing the femoral head, the superior acetabular rim, and the iliopsoas tendon. **Abbreviations:** IP, iliopsoas tendon; AC, acetabulum; FH, femoral head.

We placed the subject in the horizontal supine position, with no traction, with the hip in a neutral position. We performed the tenotomy using an ultrasound device (Sonosite Edge II, FUJIFILM Sonosite, Inc., Bothell, WA, United States) with a low-frequency convex probe (5–2 MHz) per the following technique:

- We identified the anterior superior iliac spine (ASIS) by palpation. We measured a point 4 cm distal to the ASIS and 1 cm medial to it using a 150-mm/6”, 0.05-mm, universal analog caliper (Mitutoyo Corporation, Kawasaki, Kanagawa, Japan) to determine the incision site ( Fig. 1 ).
- We positioned the probe transversely to the frontal plane. Next, we rotated the probe at 90° seeking the longitudinal positioning on the upper edge of the hip joint. This enabled the visualization of the iliopsoas muscle tendon, the upper edge of the acetabulum, and the femoral head, projecting an image called “stacking sign” ( Figs. 2 3 ).
- We proceeded to the US-guided infiltration of 0.5 mL of methylene blue. We introduced a disposable needle for regional anesthesia (of 0.7 × 88 mm/22 G × 3.5”, Spinocan, B. Braun Melsungen SE, Melsungen, Germany) at 40° of angulation, parallel and adjacent to the probe in a transverse position to the frontal plane on the upper edge of the acetabulum. This enabled the visualization of the psoas tendon and the staining of the tenotomy site alone.
- We used a 27-cm long-handled scalpel with a 5-mm thick and 3-cm long blade to make a 1-cm incision parallel and adjacent to the transducer to perform the technique ( Fig. 4 ).
- With real-time US visualization of the tenotomy site and femoral artery, vein, and nerve, we introduced the scalpel at the approach created, trying to position the blade inferior to the tendon. The tenotomy occurred with subtle movements from medial to lateral ( Fig. 5 ).
- After the percutaneous tenotomy, we performed a quadrangular flap and dissected it in planes around the scalpel to check for injured structures, structures at risk, and the technical outcome ( Fig. 6 ).
- We visually inspected and took detailed measurements of the anatomical structures adjacent to the psoas tendon and in the scalpel path, including muscles, vessels, and nerves ( Figs. 7 8 ).

**Fig. 3:**
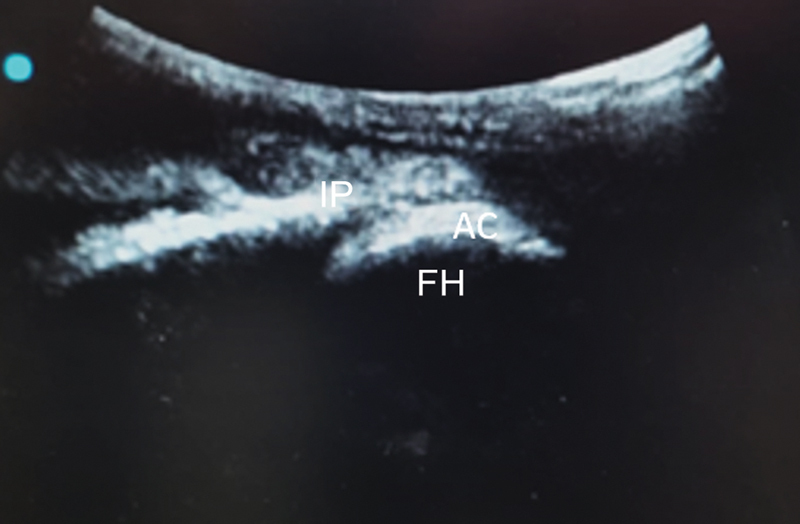
Ultrasound view of the “stacking sign” with the transducer longitudinal to the frontal plane. **Abbreviations:** IP, iliopsoas tendon; AC, acetabulum; FH, femoral head.

**Fig. 4:**
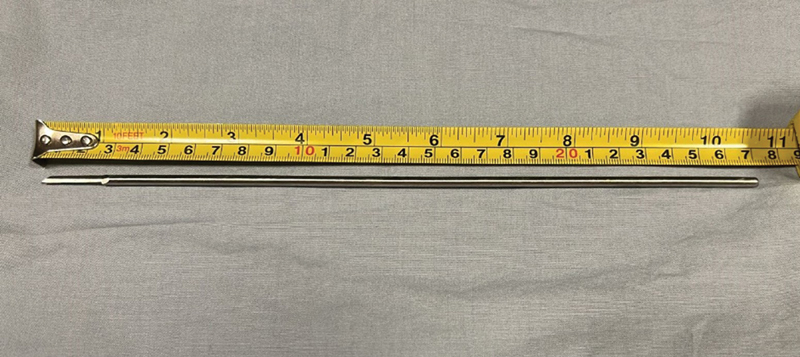
Scalpel used to perform the technique.

**Fig. 5:**
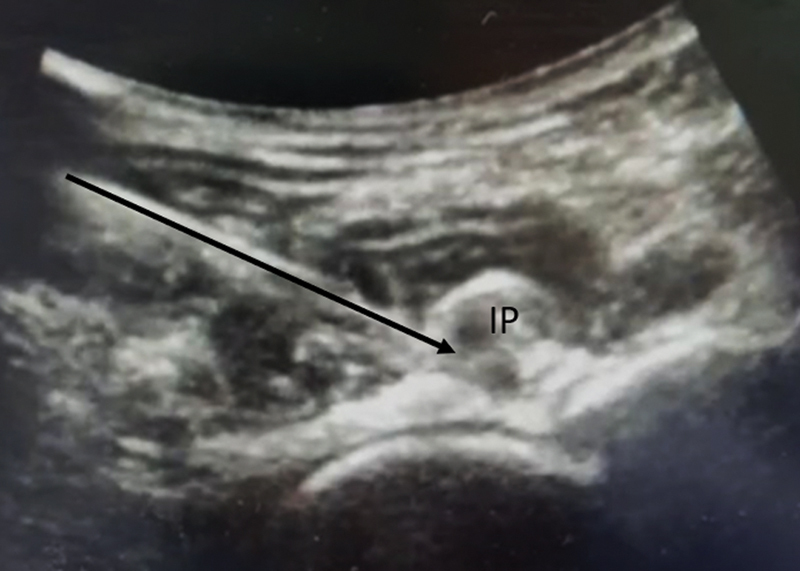
Ultrasound image of the blade path during guided iliopsoas tenotomy. **Abbreviation:** IP, iliopsoas tendon.

**Fig. 6:**
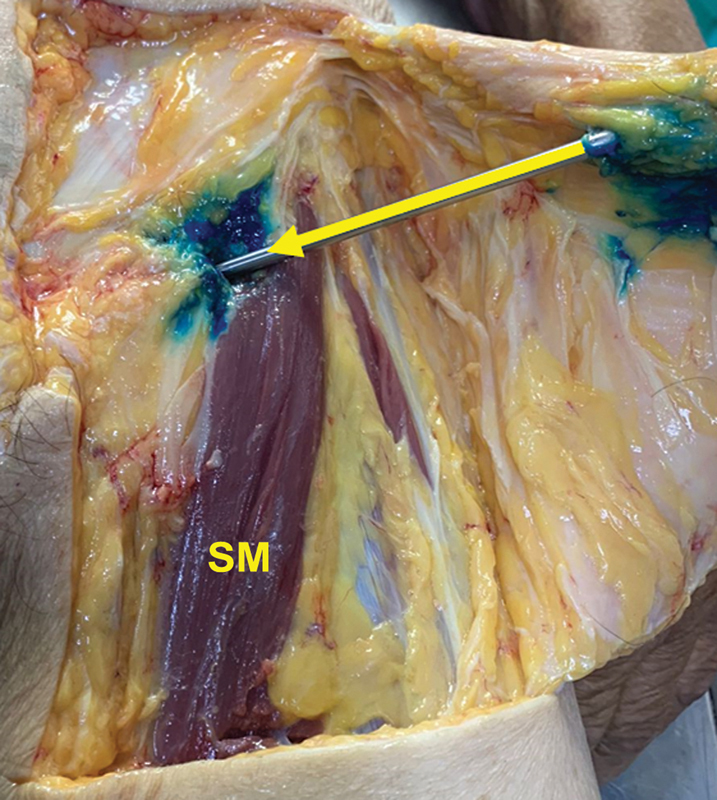
Quadrangular flap and visual inspection of the scalpel path transfixing the sartorius muscle. **Abbreviation:** SM, sartorius muscle.

**Fig. 7:**
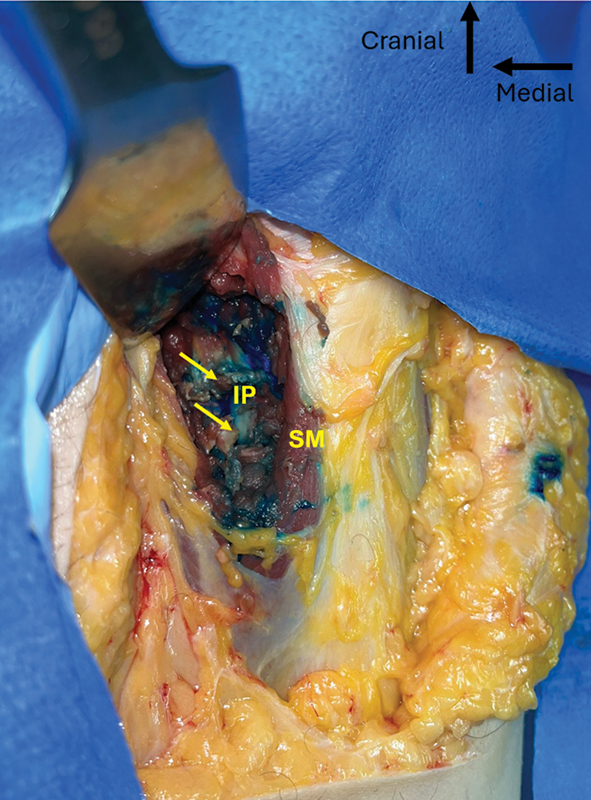
Dissection and deep inspection of the iliopsoas tenotomy. The arrows indicate the proximal and distal stumps of the tendon. **Abbreviations:** IP, iliopsoas tendon; SM, sartorius muscle.

**Fig. 8:**
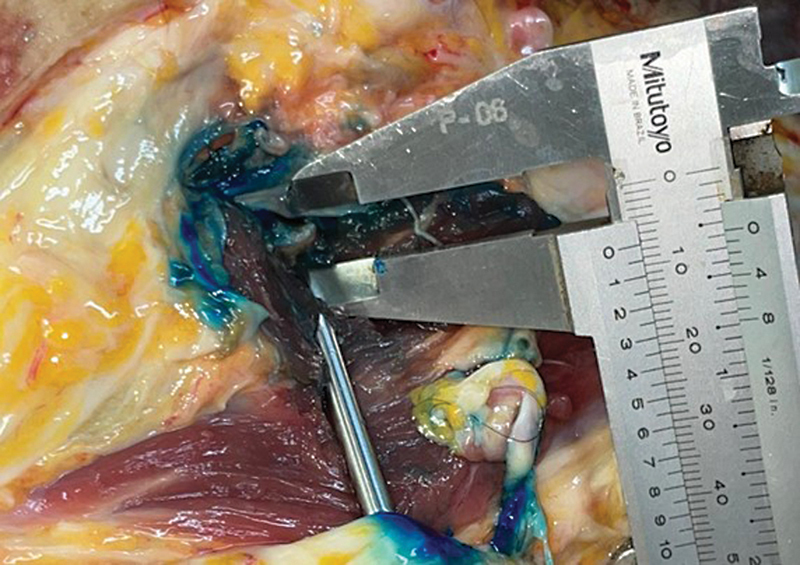
Measurement of the distance between the tenotomy site and the femoral neurovascular bundle.

### Tenotomy Analysis

To dissect the anterior region of the hip, we created a quadrangular skin flap with its apex in the middle of the medial aspect of the thigh and its base between the ASIS and the inferior gluteal fold. After dissection, we identified the ASIS, the anterior inferior iliac spine (AIIS), the inguinal ligament, the femoral neurovascular bundle, the joint capsule, the sartorius muscle, the rectus femoris muscle, and the iliopsoas muscle.

After identifying these anatomical structures, we assessed their correlations with the scalpel path, the potential injuries, and the effectiveness of the tenotomy. In addition, we measured the distance between the tenotomy site and the femoral neurovascular bundle using the same analog caliper.

## Results

We performed 20 iliopsoas tenotomies on 10 (6 male and 4 female) cadavers with ages ranging from 55 to 94 (mean: 75.2) years. Their body mass index (BMI) ranged from 12.4 to 28.7 (mean: 22.5) Kg/m ^2^ . We excluded one subject who presented an extruded bilateral inguinal hernia, which prevented the technique's performance ( Table 1 ).

**Table 1:** Anthropometric data of the study sample

Identification	Sex	Age (years)	BMI (Kg/m ^2^ )
C1	Male	61	24.2
C2	Male	67	25.7
C3	Male	71	26.2
C4	Female	85	25.9
C5	Female	89	28.7
C6	Female	55	19.3
C7	Male	58	22.1
C8	Male	88	16.4
C9	Female	94	12.4
C10	Male	84	19.2

**Abbreviations:** C, cadaver; BMI, body mass index.

**Fig. 9:**
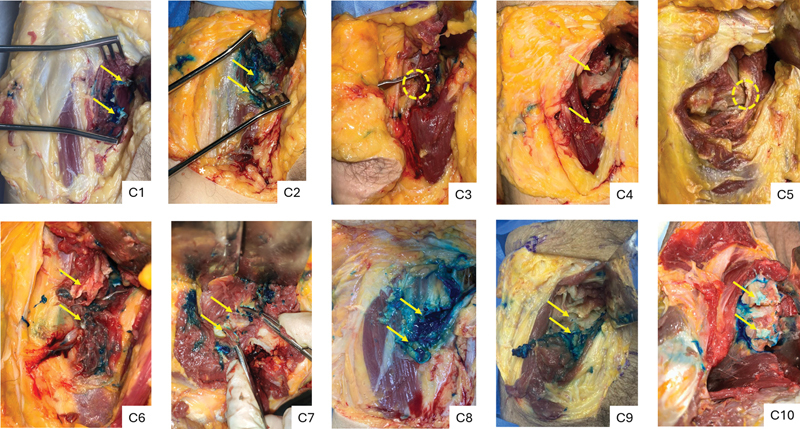
Right hips undergoing ultrasound-guided percutaneous tenotomy. The arrows indicate the distal and proximal stumps of the iliopsoas tendon, and the dotted circles show incomplete tenotomies.

Of the 20 tenotomies, 17 were total, at the level of the upper edge of the acetabulum, while 3 were partial. Among these partial tenotomies, two were performed on the subject with the highest BMI, and one, on the cadaver with the second highest BMI ( Figs. 9 10 ).

**Fig. 10:**
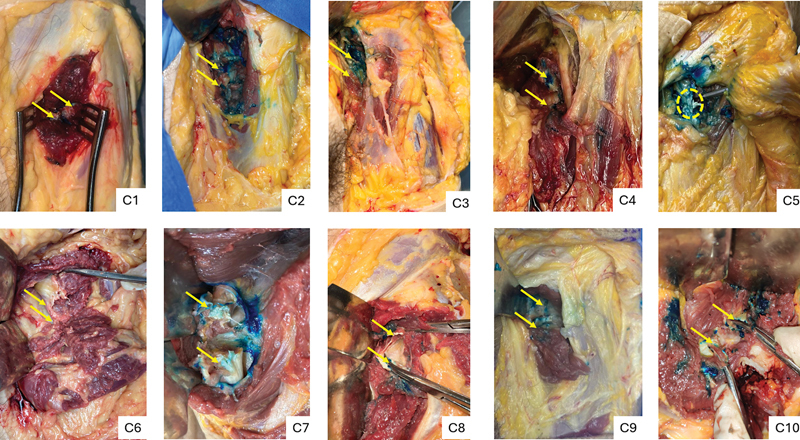
Left hips undergoing ultrasound-guided percutaneous tenotomy. The arrows indicate the distal and proximal stumps of the iliopsoas tendon, and the dotted circle shows an incomplete tenotomy.

One of the tenotomies resulted in a partial injury to the femoral nerve of the subject with the highest BMI. We observed that all tenotomies were close to the myotendinous portion and, in 14 of the 20 procedures, the blade caused a partial transfixing injury of the sartorius muscle. We evaluated the joint capsule, which showed no injuries in any procedure ( Table 2 ).

**Table 2:** Tenotomy data and analysis

Identification	Complete right-sided tenotomy	Right neurovascular bundle injury	Complete left-sided tenotomy	Left neurovascular bundle injury
C1	Yes	No	Yes	No
C2	Yes	No	Yes	No
C3	No	No	Yes	No
C4	Yes	No	Yes	No
C5	No	Yes	No	No
C6	Yes	No	Yes	No
C7	Yes	No	Yes	No
C8	Yes	No	Yes	No
C9	Yes	No	Yes	No
C10	Yes	No	Yes	No

**Abbreviation:** C, cadaver.

We measured the distance between the site where the blade was introduced and the femoral nerve, a noble structure potentially at greater risk during the procedure. The average distance was of 8.4 (range: 5–12) mm. We noted that high BMIs increased the difficulty and duration of the procedure.

## Discussion

One of the causes of anterior hip pain and difficulty in daily activities, such as climbing stairs or getting into cars, is the internal protrusion of the hip caused by the iliopsoas muscle. 2 Initially, the treatment can be conservative, with success rates of up to 50% depending on the degree of anterior extrusion of the acetabular component. When the conservative treatment fails, it may be necessary to release the iliopsoas tendon before reviewing the acetabular component. 7

The release of the iliopsoas tendon usually occurs arthroscopically, presenting good outcomes and lower risks than those of open surgeries. 7 8 9 However, this procedure requires general anesthesia and addressing a previously operated joint, which can lead to complications such as infection, leakage of the fluid infused through the tendon sheath, and other risks inherent to the technique. In addition, it is a more expensive procedure. 5 8 10 11

Seeking a less-invasive, faster, and equally-effective way to perform this release, Sampson et al. 6 were the first to report the US-guided technique. 6 Next, Johnson et al. 12 used this technique and performed the release on ten hips, without damaging noble structures. The tenotomy was incomplete in five cases. Although these authors 12 also conducted an anatomical study of the region, they did not measure the distances between the tenotomy site and the noble structures for a better assessment of the risks of this procedure.

In the present study, using a long blade instead of a needle, we achieved complete release in 17 of the 20 hips, with only 3 incomplete releases. Of these, two occurred in the cadaver with the highest BMI; this same subject suffered an incomplete injury of 4 mm to the femoral nerve. Our measurements revealed that the mean distance from the tenotomy site to the femoral nerve was of 8.4 mm. The femoral nerve injury in this single case can be attributed to an anatomical variation of the nerve, 13 and it could be avoided by direct US visualization and releasing the tendon from lateral to medial. Other than that, we observed less significant findings, such as sartorius transfixation and proximal injury to the psoas muscle.

This technique is promising, as the use of US avoids injuries to adjacent structures, such as the femoral plexus and joint capsule. In clinical studies, this technique may prove cheaper than arthroscopy, with a shorter time until the return to daily activities, and fewer complications related to anesthesia and surgical wounds.

The present study has its limitations. Since it employed cadavers, it is impossible to follow up the progression and resolution of symptoms. A single surgeon with extensive experience in US performed the releases, and the procedure may be more complex for less experienced professionals. All procedures used a scalpel with a long handle and a 3-cm blade, and we noted a greater difficulty in cadavers with higher BMIs. It is impossible to know the progression of cases with incomplete tenotomy and how devastating the femoral nerve injury would be.

Although the results cannot be completely reliable for a clinical setting due to the minimally-invasive approach, it is plausible that this technique can be safely performed with local anesthesia. Moreover, it presents lower costs than those of arthroscopy and provides a faster recovery to return to activities compared with traditional surgical procedures. Further research is required to evaluate its safety and efficacy in clinical applications.

## Conclusion

The US-guided iliopsoas muscle tendon tenotomy procedure is feasible in a cadaveric model, consistently obtaining total tendon release, except in cases of obesity, and with minimal repercussions on adjacent structures.
