# Supplementary material for: Anatomical Study of the Ultrasound-guided Percutaneous Tenotomy of the Iliopsoas Muscle Tendon in Cadavers: A Feasible Technique?
Source: Rev Bras Ortop (Sao Paulo). 2025 Apr 11;60(1):s00441790215. doi: 10.1055/s-0044-1790215 (PMC12020555; doi:10.1055/s-0044-1790215)
Supplement: Related texts

# Research article: Estudo anatômico da tenotomia percutânea do tendão do músculo iliopsoas guiada por ultrassonografia em cadáveres: Técnica viável?

**Keywords:** articulação do quadril, cadáver, quadril, tenotomia, ultrassonografia

## Abstract

**Objetivo** Avaliar a eficácia e a segurança da tenotomia percutânea do tendão do músculo iliopsoas guiada por ultrassonografia (USG) em cadáveres.

**Métodos** Realizamos um estudo anatômico e descritivo da técnica de tenotomia percutânea do tendão do músculo iliopsoas guiada por USG para repassar nossa experiência ao realizá-la e a reprodutibilidade na prática clínica.

**Resultados** Das 20 tenotomias, 17 foram totais, realizadas no nível da borda superior do acetábulo, e 3 foram parciais. Durante um dos procedimentos, houve uma lesão parcial do nervo femoral. Foram realizadas as medições da distância entre o local onde a lâmina foi introduzida e o nervo femoral, uma estrutura nobre que estaria em maior risco durante o procedimento, e a distância média foi de 8,4 mm.

**Conclusão** Os procedimentos de liberação do tendão do iliopsoas guiados por USG podem ser executados em um modelo cadavérico, pois obtém-se de forma consistente a liberação total do tendão, exceto em casos de obesidade, com mínimas repercussões em estruturas adjacentes; os procedimentos demandam aproximadamente 4 minutos para serem concluídos.

## Introdução

A artroplastia total de quadril (ATQ) é uma intervenção amplamente utilizada para restaurar a função e aliviar a dor em pacientes com diversas afeções da articulação do quadril. Entretanto, mesmo com o sucesso da ATQ, alguns pacientes continuam a sentir dor persistente no pós-operatório, muitas vezes relacionada ao atrito do tendão do músculo iliopsoas contra a borda anterior do componente acetabular. 1 2 Conhecido como impacto do psoas, resulta em inflamação, desconforto e limitação funcional significativa para o paciente, como ao subir ou descer escadas, ao entrar e sair do carro ou em situações que exijam extensão do quadril. 3 Uma das alternativas antes da revisão do componente acetabular é a realização de artroscopia com acesso intra-articular e abertura da cápsula com acesso ao tendão do músculo iliopsoas e subsequente tenotomia.

A técnica percutânea pode apresentar vantagens adicionais, como menor tempo de procedimento e somente uma incisão quando comparada às técnicas atualmente descritas, que envolvem desde acesso por artroscopia até cirurgia aberta. 4 5

Idealizou-se uma variante técnica, menos invasiva ainda, na qual o tendão do músculo iliopsoas é localizado por imagem de ultrassonografia (USG) no ponto de impacto e tenotomizado de forma guiada, como descrito previamente por Sampson et al. 6 Esta técnica é alternativa à abordagem artroscópica, que é minimamente invasiva, mas pode resultar em maiores riscos, como complicações inerentes à técnica ou extravasamento de líquido de irrigação pela bainha do iliopsoas. 5

Neste estudo, foram analisados o acesso, o potencial de risco para estruturas nobres e a efetividade da tenotomia em cadáveres. As investigações incluíram uma meticulosa avaliação da técnica utilizada durante as intervenções, com atenção especial para a minimização de danos a estruturas nobres circundantes.

O objetivo deste estudo é avaliar a eficácia e a segurança da tenotomia percutânea do tendão do músculo iliopsoas em cadáveres com o auxílio de um aparelho de USG.

## Materiais e Métodos

Trata-se de um estudo anatômico e descritivo, desenvolvido no Departamento de Ortopedia e Traumatologia da Faculdade de Ciências Médicas Santa Casa de Misericórdia de São Paulo, e executado no Serviço de Verificação de Óbitos da Capital (SVOC), da Universidade de São Paulo (USP), no qual a equipe do estudo está devidamente inscrita sob o ofício de número 18/2024, de acordo com a sua normativa; e o estudo foi aprovado pelo comitê de ética da instituição (CAAE: 76524523.4.0000.5479). Os responsáveis assinaram termo de consentimento livre e esclarecido.

O estudo incluiu uma amostra de 10 cadáveres, com 20 quadris não formolizados.

## Técnica da Tenotomia

O cadáver foi posicionado em decúbito dorsal horizontal (DDH) e sem tração, com o quadril em posição neutra. A tenotomia foi realizada com o auxílio de um aparelho de ultrassonografia (Sonosite Edge II, FUJIFILM Sonosite, Inc., Bothell, WA, Estados Unidos) com sonda convexa de baixa frequência (5–2 MHz) e conforme a seguinte técnica:

- A espinha ilíaca anterossuperior (EIAS) foi identificada por palpação. Um ponto 4 cm distal à EIAS e 1 cm para medial foi mensurado com um paquímetro analógico universal de 150 mm/6”, 0,05 mm, (Mitutoyo Corporation, Kawasaki, Kanagawa, Japão), onde será o local da incisão ( Fig. 1 ).
- A sonda foi posicionada transversalmente ao plano frontal. Depois, foi realizada a rotação de 90° da sonda para buscar um posicionamento longitudinal sobre a borda superior da articulação do quadril. Isso permitiu a visualização do tendão do iliopsoas, a borda superior do acetábulo e a cabeça femoral, projetando uma imagem denominada “sinal do empilhamento” ( Figs. 2 3 ).
- Foi realizada a infiltração com 0,5 mL de azul de metileno com o auxílio do aparelho de USG. Uma agulha descartável para anestesia regional (de 0,7 × 88 mm/22 G × 3.5”, Spinocan, B. Braun Melsungen SE, Melsungen, Alemanha) foi introduzida com 40° de angulação, paralela e adjacente à sonda, na posição transversal ao plano frontal, sobre a borda superior do acetábulo. Isso permitiu a visualização o tendão do psoas, e buscou-se corar apenas o local da tenotomia.
- Foi utilizado um bisturi de cabo longo de 27 cm com lâmina de 5 mm de espessura e 3 cm de comprimento. Foi realizada uma incisão de 1 cm paralela e adjacente ao transdutor para a realização da técnica ( Fig. 4 ).
- Com a visualização em tempo real do local da tenotomia, artéria, veia e nervo femoral por USG, o bisturi foi introduzido pela via realizada, buscando posicionar a lâmina numa posição inferior ao tendão. Com movimentos sutis de medial para lateral, a tenotomia foi realizada ( Fig. 5 ).
- Após a tenotomia percutânea, foi realizado um retalho quadrangular e dissecção por planos em volta do bisturi para a checagem das estruturas lesadas, das estruturas em risco e do resultado da técnica ( Fig. 6 ).
- Foi feita uma inspeção visual e medição detalhada das estruturas anatômicas adjacentes ao tendão do psoas e no trajeto do bisturi utilizado, como musculatura, vasos e nervos ( Figs. 7 8 ).

**Fig. 1:** Local da incisão demarcada 4 cm distalmente e 1 cm medialmente com relação à espinha ilíaca anterossuperior (EIAS). **Abreviatura:** SP, sínfise pubiana.

**Fig. 2:** Visão ultrassonográfica do “sinal do empilhamento” com o transdutor posicionado de forma transversal ao plano frontal mostrando a cabeça femoral, a borda acetabular superior e o tendão do iliopsoas. **Abreviaturas:** IP, tendão do iliopsoas; AC, acetábulo; CF, cabeça femoral.

**Fig. 3:** Visão ultrassonográfica do “sinal do empilhamento” com o transdutor posicionado de forma longitudinal ao plano frontal. **Abreviaturas:** IP, tendão do iliopsoas; AC, acetábulo; CF, cabeça femoral.

**Fig. 4:** Bisturi utilizado para a realização da técnica.

**Fig. 5:** Imagem ultrassonográfica do trajeto da lâmina na realização da tenotomia guiada do iliopsoas. **Abreviatura:** IP, tendão do Iliopsoas.

**Fig. 6:** Retalho quadrangular e inspeção visual do trajeto do bisturi transfixando o músculo sartório. **Abreviatura:** MS, músculo sartório.

**Fig. 7:** Dissecção e inspeção profunda da tenotomia do iliopsoas, e as setas apontam para os cotos proximal e distal do tendão. **Abreviatura:** MS, músculo sartório.

**Fig. 8:** Medição da distância entre o local da tenotomia e o feixe vasculonervoso femoral.

## Análise das Tenotomias

A dissecção da região anterior do quadril foi realizada com a confecção de um retalho de pele quadrangular, cujo ápice encontrava-se no meio da face medial da coxa, e a base, entre a EIAS e a prega glútea inferior. Após a dissecção, procedeu-se à identificação da EIAS, da espinha ilíaca anteroinferior (EIAI), do ligamento inguinal, do feixe vasculonervoso femoral, da cápsula articular, do músculo sartório, do músculo reto femoral e do músculo iliopsoas.

Após a identificação das estruturas anatômicas propostas, foram avaliadas suas correlações com o pertuito, as eventuais lesões e a eficácia da tenotomia. Além disso, realizou-se a mensuração da distância do local da tenotomia com o feixe vasculonervoso femoral. O mesmo paquímetro analógico foi utilizado para a medição.

## Resultados

Foram realizadas 20 tenotomias do iliopsoas em 10 cadáveres, sendo 6 do sexo masculino e 4 do sexo feminino, com idades entre 55 e 94 (média: 75,2) anos. O índice de massa corporal (IMC) dos pacientes variou de 12,4 a 28,7 (média: 22,5) Kg/m ^2^ . Um cadáver foi excluído do estudo devido à presença de hérnia inguinal bilateral extrusa, o que impede a realização da técnica ( Tabela 1 ).

**Tabela 1:** Dados antropométricos da amostra do estudo

Identificação	Sexo	Idade (anos)	IMC (Kg/m ^2^ )
C1	Masculino	61	24,2
C2	Masculino	67	25,7
C3	Masculino	71	26,2
C4	Feminino	85	25,9
C5	Feminino	89	28,7
C6	Feminino	55	19,3
C7	Masculino	58	22,1
C8	Masculino	88	16,4
C9	Feminino	94	12,4
C10	Masculino	84	19,2

**Abreviaturas:** C, cadáver; IMC, índice de massa corporal.

Das 20 tenotomias, 17 foram totais, realizadas no nível da borda superior do acetábulo, e 3 foram parciais; e destas, 2 foram realizadas no cadáver com o maior IMC, e a outra, no cadáver com o segundo maior IMC ( Figs. 9 10 ).

**Fig. 9:** Quadris direitos submetidos a tenotomia percutânea guiada por ultrassom; as setas apontam para os cotos distal e proximal do tendão do iliopsoas, e os círculos pontilhados mostram as tenotomias que foram incompletas.

**Fig. 10:** Quadris esquerdos submetidos a tenotomia percutânea guiada por ultrassom; as setas apontam para os cotos distal e proximal do tendão do iliopsoas, e o círculo pontilhado mostra a tenotomia que foi incompleta.

Durante uma das tenotomias, houve uma lesão parcial do nervo femoral, também no cadáver com o maior IMC. Observou-se que as tenotomias foram próximas à porção miotendínea em todos os casos, e, em 14 dos 20 procedimentos, houve lesão transfixante parcial do músculo sartório pela lâmina. A cápsula articular também foi avaliada, e não apresentou lesões em nenhum dos procedimentos ( Tabela 2 ).

**Tabela 2:** Dados da tenotomia e análise

Identificação	Tenotomia completa à direita	Lesão do feixe vasculonervoso direito	Tenotomia completa à esquerda	Lesão do feixe vasculonervoso esquerdo
C1	Sim	Não	Sim	Não
C2	Sim	Não	Sim	Não
C3	Não	Não	Sim	Não
C4	Sim	Não	Sim	Não
C5	Não	Sim	Não	Não
C6	Sim	Não	Sim	Não
C7	Sim	Não	Sim	Não
C8	Sim	Não	Sim	Não
C9	Sim	Não	Sim	Não
C10	Sim	Não	Sim	Não

**Abreviatura:** C, cadáver.

Foi realizada a medição da distância entre o local em que a lâmina foi introduzida e o nervo femoral, uma estrutura nobre que estaria em maior risco durante o procedimento, e a média foi de 8,4 mm, com variação de 5 a 12 mm. Constatou-se maior dificuldade e maior duração do procedimento nos cadáveres com IMC elevado.

## Discussão

Uma das causas de dor anterior no quadril e dificuldade nas atividades diárias, como subir escadas ou entrar em carros, pode ser atribuída ao ressalto interno do quadril causado pelo iliopsoas. 2 No início, essa patologia pode ser tratada de forma conservadora com sucesso em até 50% dos casos, dependendo do grau de extrusão anterior do componente acetabular. Quando não há melhora com o tratamento conservador, pode ser necessária a liberação do tendão do iliopsoas antes da revisão do componente acetabular. 7

A liberação do tendão do iliopsoas é geralmente realizada por via artroscópica, e apresenta bons resultados e menores riscos quando comparada com as cirurgias abertas. 7 8 9 Apesar disso, é um procedimento que requer anestesia geral e aborda uma articulação previamente operada, o que pode levar a complicações como infecção, extravasamento do líquido infundido pela bainha do tendão e outros riscos inerentes à técnica, além de ser um procedimento de maior custo. 5 8 10 11

Buscando uma forma menos invasiva, mais rápida e igualmente eficaz de realizar esta liberação, Sampson et al. 6 foram os primeiros a relatar a técnica que utiliza a USG como guia. Após esse relato de caso, Johnson et al. 12 utilizaram essa técnica e realizaram a liberação em 10 quadris, sem lesão de estruturas nobres. Em 5 casos, a tenotomia foi incompleta. Também foi realizado um estudo anatômico da região, 12 mas não foram medidas as distâncias entre o local da tenotomia e as estruturas nobres para que se fizesse uma melhor avaliação dos riscos desse procedimento.

Em nosso trabalho, utilizando uma lâmina longa em vez de uma agulha para a liberação, conseguimos a liberação completa em 17 dos 20 quadris, e apenas 3 tiveram a liberação incompleta. Desses, 2 foram feitas no cadáver com o maior IMC, e neste mesmo paciente ocorreu uma lesão incompleta de 4 mm do nervo femoral. Em nossos achados mensuráveis, a distância do local da tenotomia até o nervo femoral foi em média de 8,4 mm. A lesão do nervo femoral nesse único caso pode ser explicada por uma variação anatômica do nervo, 13 e pode ser evitada pela visualização direta pela USG e pela realização da liberação do tendão de lateral para medial. Fora isso, obtivemos achados menos significativos, como a transfixação do sartório e a lesão muscular proximal do músculo psoas.

Esta técnica é promissora, pois a USG nos permite evitar lesão de estruturas adjacentes, como o plexo femoral e a cápsula articular. Estudos clínicos talvez demonstrem que essa técnica tem um custo menor do que o da artroscopia, com menor tempo até o retorno às atividades diárias e menores complicações relacionadas à anestesia e à ferida operatória.

Este estudo tem suas limitações, uma vez que foi realizado em cadáveres, sendo impossível acompanhar a evolução e a resolução dos sintomas. As liberações foram realizadas somente por um operador com ampla experiência no uso da USG, e elas podem ser mais difíceis para operadores menos experientes. Todos os procedimentos foram realizados usando um bisturi de cabo longo e lâmina de 3 cm, e constatou-se maior dificuldade nos cadáveres de maior IMC. Não é possível saber como seria a evolução dos casos em que a tenotomia foi incompleta, e quão devastadora seria a lesão do nervo femoral.

Embora os resultados não possam ser totalmente fidedignos para um ambiente clínico, devido à abordagem minimamente invasiva do procedimento, é plausível que essa técnica seja realizada de forma segura com anestesia local, apresente custos inferiores em comparação com os da artroscopia e proporcione uma recuperação mais rápida para o retorno às atividades, se comparada aos procedimentos cirúrgicos tradicionais. Pesquisas adicionais são necessárias para avaliar a segurança e eficácia em aplicações clínicas.

## Conclusão

O procedimento de tenotomia do tendão do músculo iliopsoas, orientado por USG, pode ser executado em modelo cadavérico, pois obtém-se de forma consistente a liberação total do tendão, exceto em casos de obesidade, com mínimas repercussões em estruturas adjacentes.
